# Bioinformatics for Inosine: Tools and Approaches to Trace This Elusive RNA Modification

**DOI:** 10.3390/genes15080996

**Published:** 2024-07-29

**Authors:** Enrico Bortoletto, Umberto Rosani

**Affiliations:** Department of Biology, University of Padova, 35131 Padova, Italy; enrico.bortoletto@unipd.it

**Keywords:** inosine, ADAR, RNA editing, A-to-I editing, bioinformatics

## Abstract

Inosine is a nucleotide resulting from the deamination of adenosine in RNA. This chemical modification process, known as RNA editing, is typically mediated by a family of double-stranded RNA binding proteins named Adenosine Deaminase Acting on dsRNA (ADAR). While the presence of ADAR orthologs has been traced throughout the evolution of metazoans, the existence and extension of RNA editing have been characterized in a more limited number of animals so far. Undoubtedly, ADAR-mediated RNA editing plays a vital role in physiology, organismal development and disease, making the understanding of the evolutionary conservation of this phenomenon pivotal to a deep characterization of relevant biological processes. However, the lack of direct high-throughput methods to reveal RNA modifications at single nucleotide resolution limited an extended investigation of RNA editing. Nowadays, these methods have been developed, and appropriate bioinformatic pipelines are required to fully exploit this data, which can complement existing approaches to detect ADAR editing. Here, we review the current literature on the “bioinformatics for inosine” subject and we discuss future research avenues in the field.

## 1. Inosine and ADAR Protein Discovery

**Figure 1:**
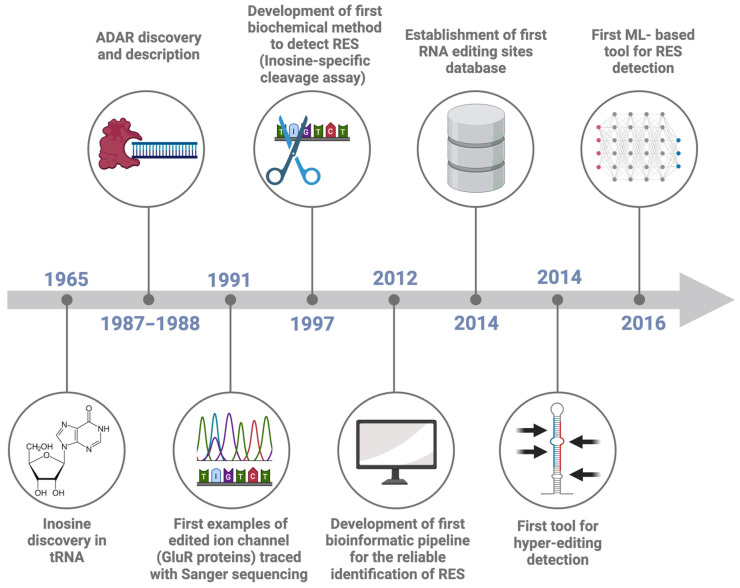
Timeline of RNA Editing Sites (RES) detection. The Figure reports the most important events in the history of advancing in RNA editing site detection, namely: inosine discovery in tRNA (1965, [14]), the discovery of the protein responsible for RNA editing (1987–1988, [17,18,19]), the first example of edited ion channel (1991, [21]), the development of a first biochemical method to detect inosine (1997, [23]), the development of a first bioinformatic pipeline for the reliable identification of RES (2012, [24]), the creation of the first RES database (2014, [25]), a first tool for hyper-editing detection (2014, [26]) and a first machine learning based tool for RES detection (2016, [27]).

The presence of naturally occurring RNA modifications was first reported in 1960 [1,2,3], with over 170 distinct RNA modifications identified so far [4]. These modifications can be present in different types of RNA and can influence RNA stability and structure, and the interactions between RNA and cellular or viral proteins [5,6]. Indeed, RNA modifications play crucial roles in regulating RNA metabolism by affecting RNA processing, localization, and translation into proteins, particularly when messenger RNAs (mRNAs), transfer RNAs (tRNAs), and ribosomal RNAs (rRNAs) are impacted [7]. Accordingly, RNA modifications can be seen as a bridge linking gene transcription to protein synthesis, thereby influencing cell functioning. Several tools and methods have been developed to trace the different modifications occurring in RNA molecules. Most of the efforts were in the study of N^1^-Methyladenosine (m^1^A), 5-Methylcytosine (m^5^C), 5-Methyluridine (m^5^U), N6-Methyladenosine (m6A), N^6^,2′-O-dimethyladenosine (m^6^Am), 7-methylguanosine (m^7^G), pseudouridine (Ψ), and 2′-O-Methylation (Nm) (Table 1, with more details in Supplementary Table S1). RNA editing is a post-transcriptional activity, defined as the insertion, deletion or chemical modification of ribonucleotides in target RNA molecules [8,9,10]. Compared to the eukaryotic RNA splicing and polyadenylation processes, RNA editing is less frequent but still pivotal in modifying the function and stability of RNAs, and its involvement in health and disease is documented by several lines of evidence in humans [11,12,13]. The Adenosine (A) to Inosine (I) deamination (known as A-to-I editing) represents one of the most frequent RNA modifications in metazoans [10]. Inosine was first discovered in tRNAs in 1965 [14] (Figure 1), and later, in 1966, it was shown that inosine could pair with multiple nucleotides, de facto expanding the flexibility in the genetic code during protein synthesis [10] and therefore opening the avenue for the study of RNA editing [15] and modification, a field dubbed epitranscriptomics [16].

The protein responsible for the enzymatic deamination of adenosine into inosine was initially described in 1987 as a protein capable of unwinding the RNA duplexes in *Xenopus laevis* embryo, thus impairing the inhibition of mRNA translation by the injection of a complementary antisense RNA [17,18]. One year later, further characterization of this protein demonstrated its capability to modify the RNA enzymatically (Figure 1) [19]. The protein, called Double-stranded RNA-specific adenosine deaminase (abbreviated into DRADA, ADAR or dsRAD), was then deeply studied, revealing a pivotal role in maintaining the organism homeostasis [10,20,21,22].

ADAR proteins are characterized by a variable number of dsRNA binding domains and by a deaminase domain. The dsRNA binding domain allows the binding of ADAR to dsRNA structures, whereas the deaminase domain performs the deamination of the target adenosine [28]. In humans, three ADAR paralogs are present. Consistently, in the evolution of animals, ADAR1 generally possesses one or two extra domains, enabling binding to Z-DNA/Z-RNA structures [28]. As for the three mammalian ADAR genes (ADAR1-3), ADAR1 and ADAR2 are expressed throughout the body, but ADAR1 is generally more expressed than ADAR2 and accounts for the majority of the editing activity. Conversely, ADAR3, which is catalytically inactive, is exclusively expressed in the brain [29,30]. 

At this point, it becomes essential to distinguish between two different types of ADAR-mediated RNA modifications. The first, called single-base editing, results in one or a few edited adenosines per RNA molecule. In contrast, the second type of modification, called hyper-editing, involves multiple A-to-I modifications on the same molecule [26,31,32]. Since the inosine pattern of hydrogen bonds is like that of guanosine, inosine is read as guanosine during protein translation [33,34]. Therefore, A-to-I editing has the potential to alter the coding capacity of mRNAs, in some cases with critical biological consequences [35]. Actually, A-to-I editing of GluR2 transcripts is needed for the normal development of the nervous system in mice and zebrafish [35,36], and perturbed A-to-I editing has been associated with behavioral changes in the fruit fly [37]. In the squid nervous system, extensive A-to-I editing mainly occurs in the giant axons rather than in the cell body—indicating tissue-specific RNA editing—where it can modulate the microtubule motor protein function in response to environmental changes [38,39]. Moreover, A-to-I editing contributes to germline cell integrity by preventing the spread of repetitive transposable elements, such as Alu and SINE in humans and mice, respectively [22,40,41]. Overall, these studies have revealed the importance of ADAR editing in physiology, development and disease, making a better understanding of the occurrence and extension of this phenomenon across the evolution of metazoans necessary.

## 2. The Development of Inosine Detection Methods

Since 1997, several methods have been developed to trace the enzymatic activity of ADAR. For the sake of clarity, we divided all these methods into direct or indirect ones, according to whether they directly detected inosine or whether the inosine detection was performed indirectly (Table 2).

Direct inosine detection methods include low- to medium-throughput approaches, such as two-dimensional, thin-layer chromatography (2D-TLC) [42], inosine-specific cleavage assays [23] and mass spectrometry [43]. In addition, some specific protocols were developed to retrieve the A-to-I modifications in RNA, such as DARTS [44], ICE-seq [45], ALES [46] and hEndoV-seq [47]. Regarding the indirect methods, the most used consists of tracing the inosine footprint using Sanger or high-throughput sequencers. The classical Sanger sequencing method can be adapted for inosine detection by reverse transcribing RNA into cDNA [48]. During reverse transcription, inosine pairs with cytidine and is read as guanosine, resulting in A-to-G mismatches in the sequencing data [21]. Next-Generation Sequencing (NGS) offers both the possibility to generate high-throughput data and the single-nucleotide resolution necessary to detect RNA modifications, following the same principle applied to Sanger sequencing. Although NGS approaches can provide comprehensive and detailed insights about RNA editing, they require extensive data analysis efforts [24]. 

## 3. Strategies for Genome-Wide Identification of ADAR-Mediated RNA Editing Sites

Inosine detection methods, particularly indirect ones, present several challenges in the computational detection of genuine RNA Editing Sites (RES) through transcriptional noise. This is the case of massive RNA sequencing data, where the high-throughput output of modern sequencers coupled with an imperfect detection of nucleotides easily introduces confounding factors (i.e., noise), hampering a straightforward detection of low-frequency modifications. False positive RES detection can lead researchers to incorrect conclusions about editing frequencies and their biological implications. Both improving sequencing accuracy and depth as well as validating potential editing sites with independent methods can reduce the impact of false positives. In addition, RNA sequences, especially mRNAs, can contain repetitive elements that complicate the alignment of sequencing reads to reference genomes. Misalignment can lead to incorrect identification of editing sites, leading to both false positives and false negatives. To overcome such a limitation, the use of alignment software optimized for RNA-seq data and the adjustment of alignment parameters to be more stringent with mismatches and gaps can be adopted. Finally, a high-quality, well-annotated reference genome is crucial for accurate mapping of sequencing reads and the subsequent identification of RES. Incomplete or incorrectly-assembled genomes can lead to erroneous conclusions about the presence and extent of RNA editing.

To perform a correct RNA editing analysis, all analytical steps should be planned appropriately. The main steps are file preprocessing, RES detection and RES filtering.

In this review, we discussed the published resources that can facilitate setting up the best possible pipeline for different objectives and organisms, including non-model species lacking a reference genome.

### 3.1. File Preprocessing

**Figure 2:**
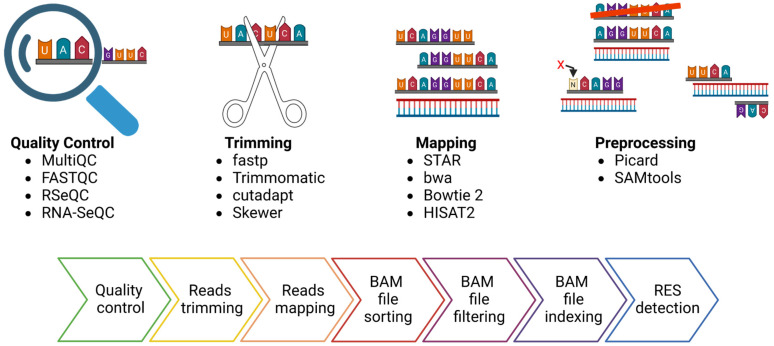
FASTQ files preprocessing for the detection of RNA Editing Sites (RES). In the upper part, the picture depicts the four most essential steps in preprocessing FASTQ files for RES detection and some available tools for each step. In the lower part of the figure, a more detailed description of the bioinformatic pipeline steps is presented.

Starting from the sequencer outputs, usually consisting of files in FASTQ format, the RES are traced by performing the following steps: reads quality control and trimming, alignment of the reads to the reference genome and preprocessing of the alignment file (Figure 2). The read quality check and trimming steps are not mandatory, but their application will improve the mapping rate and the alignment quality, two elements that can affect the detection of RES. For these two steps, several tools can be used, such as FASTQC (https://github.com/s-andrews/FastQC (accessed on 1 June 2024)), RSeQC [49] or RNA-SeQC [50], as well as MultiQC [51], a versatile tool designed to create a single comprehensive report by aggregating output from multiple tools applied on several samples. The manual inspection of the outputs of these tools facilitates the identification of global trends and biases in the raw and/or trimmed sequencing data. Similarly, there are multiple tools for data trimming, including fastp [52], Trimmomatic [53], cutadapt [54] and Skewer [55]. The main goal of data trimming is to remove all the remaining sequencing adapters and low-quality bases, which can impair the detection of RES.

As regards the mapping step, there are a variety of mapping tools and algorithms [56]. Several publications have evaluated the performance of the most commonly used mapping tools in terms of speed, accuracy, and usage of computation resources [57,58,59,60]. A recent publication confirmed that using different tools can be critical for the accuracy and resolution of the analysis [57]. Indeed, they analyzed a Genotype-Tissue Expression RNA-seq experiment from the human cerebellum (run accession ID SRR607967), showing that the number of detected sites varied depending on the aligner used. For example, the sequence alignment carried out with STAR resulted in the lowest false positive rate, suggesting its higher performance. An essential parameter regarding the mapping step is to keep only the uniquely mapping reads, avoiding the reads that map in more than one genomic location [57,58]. After the alignment, the resulting SAM file needs to be converted into BAM format, and before using the tools dedicated to RES detection, following some steps are essential to obtain reliable results. The GATK best practices provide a complete pipeline for the BAM preprocessing [61,62]. In this case, the most important steps include the BAM file sorting, the removal of duplicates and the reads filtering, keeping the mapped and properly paired reads with a minimum alignment quality decided by the user (for example, 20). Picard (https://github.com/broadinstitute/picard (accessed on 1 June 2024)) and SAMtools [63] are necessary for these steps (Figure 2).

### 3.2. Detection of RNA Editing Sites

**Figure 3:**
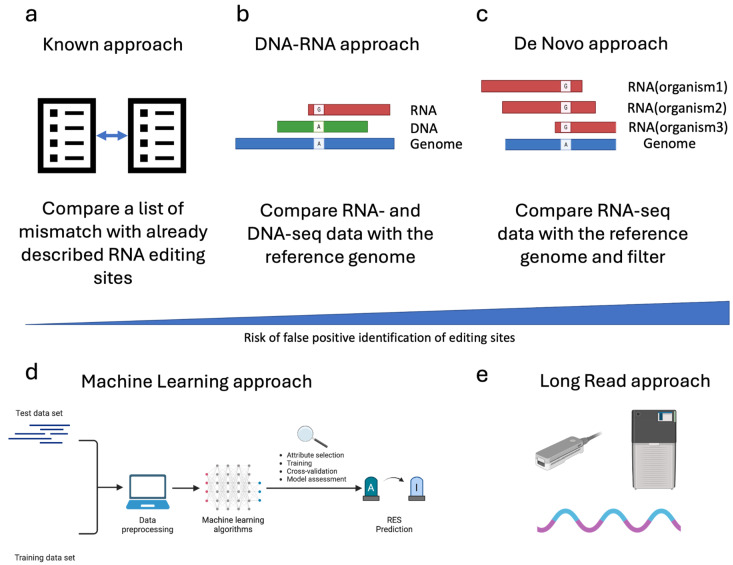
Strategies used to trace RNA Editing Sites (RES). This figure reports the most common strategies employed to trace RNA editing sites. In the case of Illumina sequencing data, in addition to the approach described based on REDItools nomenclature (Known panel (**a**), DNA-RNA panel (**b**) and De Novo panel (**c**)), an indicative bar showing the risk of false positive rate associated with the different approaches is depicted. Moreover, the most innovative methods still under development for the trace of RES are shown (the Machine learning and long-read approaches, panel (**d**) and panel (**e**), respectively).

Several strategies can be applied to detect RES. One of the most straightforward approaches is comparing a list of putative editing sites with the already described ones. This approach (named “Known”, Figure 3a) requires a list of validated editing sites, which, so far, are available only for a few species and can be found in the REDIportal database [64]. The most used tool for detecting “Known” sites is REDItools, which has one dedicated module [31].

A second approach, called “DNA-RNA”, requires the sequencing of RNA and DNA from the same individual (Figure 3b). The comparison of Single Nucleotide Variations (SNVs) by matching DNA- and RNA-seq data with the reference genome allows the correct filtering out of pre-existing (genomic) SNVs, thus lowering the false positive rate. Various tools have been developed adopting different algorithms and statistical methods to maximize the accuracy of the analysis. Additionally, editing sites can be traced to “De Novo” by exploiting RNA-seq data only. In this case, the filtering step and the statistical approach are crucial for lowering the false positive rate (Figure 3c). Some of the most used tools for RES detection in Illumina sequencing data are summarized in Table 3.

In recent years, due to the expansion of the Machine Learning algorithm (ML), some machine learning techniques have also been developed to detect RES (Figure 3d).

RDDpred [27] is one of the first tools designed using an ML approach to predict RNA editing sites from RNA sequencing data. It employs a Random Forest algorithm to accurately distinguish between genuine RNA editing events and false positives. The tool constructs condition-specific training datasets by incorporating data from established RNA-editing databases. Additionally, RDDpred utilizes the Mapping Errors Set method to identify regions prone to alignment errors. 

RED-ML [70] leverages information from various features and the properties of RES to make predictions using a logistic regression classifier for genome-wide RES identification. If DNA-seq data are available, SNPs can be specified and included in the analysis. This tool is limited to human RNA-seq data and can only identify sites with relatively high editing levels.

DeepRed [71] employs deep and ensemble learning to identify genuine RES from a list of traced SNVs. Thus, it can help validate a list of putative RNA editing sites.

Finally, the advent of long-read sequencing technology, such as Pacific Biosciences (PacBio) and Oxford Nanopore Technology (ONT), opened the avenue for new tools to exploit the potential of these novel types of data (Figure 3e). In particular, the possibility of sequencing native RNA by the direct RNA (dRNA) protocol of ONT theoretically makes the direct detection of inosine in RNA molecules possible.

Dinopore [72] is a deep learning-based tool designed to detect RNA editing sites, specifically A-to-I editing, using nanopore direct RNA sequencing data. 

DeepEdit [73] is a cutting-edge tool designed to detect A-to-I RNA editing events using nanopore direct RNA sequencing data. This tool leverages a fully connected neural network model to analyze the raw electrical signals generated during nanopore sequencing.

L-GIREMI [74] is another tool for detecting and analyzing RNA editing sites within long-read RNA-seq data. It adeptly manages sequencing errors and read biases and demonstrates high accuracy when applied to PacBio RNA-seq data.

Overall, both the ML and long-reads approaches present some limitations; namely, both strategies require good bioinformatic knowledge and considerable computational effort. In addition, these techniques are accurate for species with well-curated reference genomes, whereas they could be somewhat inaccurate if the reference genome used is not of high-quality. Moreover, for ML algorithms, the training sets mostly derive from model organisms, lowering the sensitivity and accuracy if applied to phylogenetically distant species.

### 3.3. RNA Editing Sites Filtering

**Figure 4:**
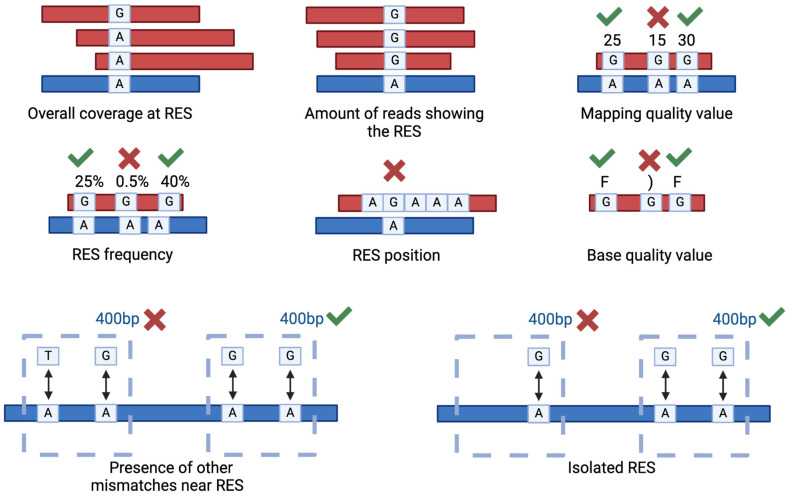
Main filtering criteria used to select genuine RNA Editing Sites (RES). The picture illustrates the main parameters used to filter the RES. The parameters include the overall coverage at RES, the number of reads displaying the RES, the associated mapping quality value, the frequency and position of the RES and the base quality values. Low quality values are, as examples, 15 or “)”, good quality ones are 30 or “F”. In addition, two strong filters are represented by the relative position of the identified RES compared to other putative RES or different types of mismatches.

Several parameters can be used to filter the putative RES in order to increase the resolution and the accuracy of the analysis (Figure 4). The most used parameters are read coverage, amount of reads supporting the RES, mapping quality, RES frequency and position. As an example, it is a good practice to exclude RES occurring in homopolymeric regions [58]. Sometimes, these parameters are insufficient for obtaining an acceptable false positive rate. For instance, in the case of zebrafish and Xenopus sp., even if using matching DNA- and RNA-seq data, only with additional filters were the authors able to identify high-quality RES [75,76]. These additional filters are based on the sequence position of the putative RES in comparison to other putative RES or to different types of mismatches [75]. Specifically, since in most of the cases the editing sites are clustered together, the RES were excluded from the results if another non-editing compatible mismatch was traced in a 400 bp genomic window, and if the RES was isolated (no other A-to-G variations in a 400 bp genomic window), then the genomic window can be adapted to match specific organisms [75].

## 4. RNA Editing Indexes

RNA editing indexes are essential for rapidly evaluating the extension of RNA editing in a given sample. Indeed, they provide the quantification of RNA editing events. Considering that most of the RNA editing in humans occurred in Alu repeats [57,58,77] and that ADAR targets the majority of Alu adenosines to some extent, one of the first RNA editing indexes was developed by considering the RES in the Alu repeats. The so-called Alu Editing Index (AEI), developed in 2019, is based on the ratio of the number of A-to-G mismatches to the total coverage of adenosines (that is, the sum of the number of A-to-G RNA-DNA mismatches and A-A matches in these regions) [78]. Correctly tracing the RNA editing sites requires considerable computation time and power, which is why the main advantage of AEI is that it allows rapid estimation of the editing level, as well as the considering of a large number of samples. However, since Alu repeats are primate-exclusive, this index cannot be used for other species as it is. However, modification of this index was proposed in order to investigate RNA editing in zebrafish by modifying the repeat type [75]. Further modification will allow us to exploit the RNA editing index in other species.

Another index is represented by the overall editing, defined as the total number of reads with G at all known editing positions over the number of all reads covering the positions, without imposing specific sequencing coverage criteria [57]. It can be calculated using REDItools tables. Nevertheless, this index requires performing the RES calling and a database containing described RES, making it inapplicable to species not covered in these databases.

Finally, the overall editing calculated at recoding positions, namely the editing events located in coding genomic regions, is referred to as the recoding index (REI) [79]. This metric, used to investigate the activity of ADAR2, can be calculated using REDItools tables and a list of recoding sites from a RES database. 

## 5. Conclusions

ADAR-mediated RNA editing is an essential biological process that can result in the alteration of protein sequences or in the modulation of mRNAs, and its analysis can contribute to improving our understanding of organisms’ homeostasis, disease and development processes. Understanding the connection between RNA editing and the fate of RNA in different species and conditions will probably untangle intimate regulative processes, which can be evolutionary conserved or species-specific. Because RNA editing can impact various layers of cellular regulation, integrating different types of omics data (such as genomics, transcriptomics, proteomics, and metabolomics) could provide a comprehensive view of the functional consequences and regulatory networks promoted by ADAR enzymatic and non-enzymatic functions. The integration of transcriptomic and genomics data has been widely used to distinguish between real RES and genomic mutations. In this context, proteomic data could provide an additional layer of validation, revealing the consequences of ADAR editing on protein synthesis. However, the integration of these omics requires sophisticated bioinformatic tools and statistical methods to manage and interpret the data appropriately. Therefore, despite its potential, multi-omics integration faces challenges such as data heterogeneity, the need for large sample sizes to achieve statistical power and computational complexity of data analysis.

Appropriate bioinformatic pipelines are needed to fully exploit datasets produced with existing technologies, as well as with novel emerging technologies able to provide a single-base resolution of any kind of RNA modification. Undoubtedly, applying neural networks and AI-based algorithms can contribute to developing novel tools. However, the requirement of an appropriate training dataset is the current challenge for their broad application. At the same time, the advancement of the accuracy of long-read sequencing and of protocols to sequence native RNA molecules could drastically contribute to the detection of inosine.

## Figures and Tables

**Table 1:** List of the most studied RNA modifications with the tools developed for their computational detection. For each tool, the detected modification, the methodology used, the extended reference and the reference number are reported in Supplementary Table S1.

RNA Modification	Tool
m^1^A	RAMPred, iRNA-3typeA, DeepPromise, MSCAN, Rm-LR, iMRM
m^5^C	iRNAm5C-PseDNC, iRNA- PseColl, PEA-m5C, pM5CS-Comp-mRMR, RNAm5Cfinder, RNAm5CPred, DeepMRMP, iRNA-m5C, m5CPred-SVM, MSCAN, Rm-LR, iMRM
m^5^U	iRNA-m5U, m5UPred, m5U-SVM, MSCAN, Rm-LR
m^6^A	iRNA-3typeA, iRNA-Methyl, m6Apred, M6ATH, RNA-MethylPred, TargetM6A, pRNAm-PC, RNAMethPre, AthMethPre, M6A-HPCS, SRAMP, MethyRNA, RAM-ESVM, RAM-NPPS, iMethyl-STTNC, iRNA(m6A)-PseDNC, HMpre, M6APred-EL, BERMP, M6AMRFS, RFAthM6A, DeepM6APred, Gene2Vec, FunDMDeep-m6A, m6A-NeuralTool, DNN-m6A, WHISTLE, Adaptive-m6A, DL-m6A, m6A-Finder, m6A-TCPred, DeepPromise, MSCAN, Rm-LR, iMRM
m^6^Am	MSCAN, Rm-LR
m^7^G	MSCAN, iRNA-m7G, m7GPredictor, XG-m7G, m7G-IFL, m7G-DLSTM, THRONE, TMSC-m7G, Moss-m7G
Ψ	MSCAN, iMRM, PPUS, iRNA-PseU, PseUI, iPseU-CNN, iPseU-NCP, EnsemPseU, PIANO, PSI-MOUSE, XG-PseU, RF-PseU, NanoPsu, Penguin
Nm	Rm-LR, iRNA-2OM, NmSEER, iRNA-PseKNC(2methyl), NmSEER V2.0, DeepOMe, NmRF, BERT2OME, i2OM, H2Opred

**Table 2:** Description of the direct and indirect methods to detect inosine in biological samples. The name, type, a brief description, and known limitations are reported for each method.

Method	Type	Description	Limitations
Thin-Layer Chromatography (2D-TLC) [42]	Direct	Detect RNA modifications by separating nucleotides based on their mobility in a solvent. RNA is partially digested into oligonucleotides, labelled with ^32^P and separated via 2D-TLC. Nucleotides are identified by comparing their retardation factors to standards and quantified by measuring radioactivity.	-Low throughput and labor-intensive. -Requires radioactive labeling, which involves safety and disposal issues. -Limited sensitivity and resolution compared to modern high-throughput methods.
Inosine-Specific Cleavage Assays [23]	Direct	This method involves treating RNA with chemicals or enzymes that specifically cleave at inosine sites. For instance, inosine-specific ribonucleases can be used to cut RNA at inosine residues, allowing for the identification of inosine locations.	-Limited to specific cleavage sites, potentially missing some inosine modifications. -Requires precise enzymatic or chemical conditions, which can be challenging to optimize. -Not suitable for high-throughput analysis.
Mass Spectrometry (MS) [43]	Direct	Identifies and quantifies inosine by analyzing the mass-to-charge ratio of RNA fragments.	-Can be complex and time-consuming to prepare samples and interpret results. -Sensitivity can be an issue for low-abundance modifications.
DARTS [44]	Direct	Allows the concurrent quantification of A-to-I editing and m^6^A modifications at the same sites in RNA.	-Limited to single-site analysis and cannot be used for transcriptome-wide analysis of RNA modifications.
ICE-seq [45]	Direct	Biochemical method useful for identifying inosines based on cyanoethylation combined with reverse transcription and RNA-seq.	-Requires chemical modification of RNA, which can be technically challenging.
ALES [46]	Direct	The ALES method detects RNA editing by modifying inosine to N1-cyanoethylinosine, which causes reverse transcription to stall at the edited site. This stalling is then measured using real-time quantitative PCR, allowing precise identification and quantification of RNA editing.	-ALES method is not capable of transcriptome-wide mapping of inosine sites.
hEndoV-seq [47]	Direct	This approach blocks the RNA terminal 3′OH using 3′-deoxyadenosine. The hEndoV protein then specifically cleaves inosine sites, creating new terminal 3′OH ends. These new ends can be identified through sequencing analysis, allowing site-specific inosine detection in RNA.	-Limited throughput, not ideal for large-scale studies.
Sanger Sequencing [48]	Indirect	Detects A-to-G mismatches after reverse transcribing RNA to cDNA.	-Low throughput and labor-intensive compared to next-generation sequencing -Limited sensitivity, especially for low-frequency editing events.
Next-Generation Sequencing (NGS) [24]	Indirect	High-throughput sequencing of cDNA. A-to-G transitions are then traced with a specific bioinformatic pipeline.	-Requires significant computational resources and bioinformatic expertise -Potential for sequencing errors and alignment ambiguities, leading to false positives.
Nanopore direct RNA sequencing	Direct	Direct sequencing of native RNA, inosine can be directly detected from the raw electric signals.	-Lack of an inosine-dedicated basecaller.

**Table 3:** Description of the different tools useful for tracing RES in Illumina sequencing data. The name, input datasets accepted and a brief description are reported for each tool considered.

Tool Name	Input Datasets	Description
REDItools [31]	DNA-RNA RNA	REDItools provides a nice output table and a series of accessory scripts for annotating and filtering the resulting RES.
RES-Scanner [65]	DNA-RNA	RES-Scanner uses a combination of different statistical models for homozygous genotype calling and filters to remove potential false-positive RES, also providing an associated *p*-value.
JACUSA [66]	DNA-RNA RNA	JACUSA is designed with user-friendliness in mind. It offers a streamlined process for detecting RNA editing events by exploiting a robust statistical approach.
RESIC [67]	DNA-RNA RNA	RESIC allows the exclusion of polymorphism sites so as to increase reliability based on DNA-seq, ADAR-mutant RNA-seq datasets or SNP databases.
GIREMI [68]	RNA	GIREMI can integrate biological replicates into one dataset for higher accuracy in RNA editing detection. Its algorithm is based on mutual information between editing sites in RNA-seq data and SNPs and is thus suitable only for diploid genomes.
SPRINT [69]	RNA	SPRINT can identify RNA editing sites, without the need to utilize SNP databases, by clustering RES and SNP duplets based on their distinctive and unique distribution.

## Data Availability

Not applicable.

## Supplementary Materials

The following supporting information can be downloaded at: https://www.mdpi.com/article/10.3390/genes15080996/s1, Supplementary Table S1. PTM detection tools. For each tool, the targeted RNA modifications, the algorithm used, the year of release and the associated publication are reported [80,81,82,83,84,85,86,87,88,89,90,91,92,93,94,95,96,97,98,99,100,101,102,103,104,105,106,107,108,109,110,111,112,113,114,115,116,117,118,119,120,121,122,123,124,125,126,127,128,129,130,131,132,133,134,135,136,137,138,139,140,141,142,143,144,145,146,147,148,149,150,151,152,153,154,155,156,157].
